# Polymorphisms in Genes Encoding Potential Mercury Transporters and Urine Mercury Concentrations in Populations Exposed to Mercury Vapor from Gold Mining

**DOI:** 10.1289/ehp.1204951

**Published:** 2012-10-09

**Authors:** Karin Engström, Shegufta Ameer, Ludovic Bernaudat, Gustav Drasch, Jennifer Baeuml, Staffan Skerfving, Stephan Bose-O’Reilly, Karin Broberg

**Affiliations:** 1 Department of Laboratory Medicine, Division of Occupational and Environmental Medicine, Lund University, Lund, Sweden; 2 United Nations Industrial Development Organization, Vienna, Austria; 3 Institute of Forensic Medicine, Ludwig Maximilians University, Munich, Germany; 4 Institute of Public Health, Medical Decision Making and Health Technology Assessment, UMIT–University for Health Sciences, Medical Informatics and Technology, Hall in Tirol, Austria; 5 Institute and Outpatient Clinic for Occupational, Social and Environmental Medicine, University Hospital Munich, Munich, Germany

**Keywords:** gold mining, inorganic mercury, LAT1, MDR1, MRP1, OAT1, OAT3, SLC3A2, SLC22A6, SLC22A8

## Abstract

Background: Elemental mercury (Hg^0^) is widely used in small-scale gold mining. Persons working or living in mining areas have high urinary concentrations of Hg (U-Hg). Differences in genes encoding potential Hg-transporters may affect uptake and elimination of Hg.

Objective: We aimed to identify single nucleotide polymorphisms (SNPs) in Hg-transporter genes that modify U-Hg.

Methods: Men and women (1,017) from Indonesia, the Philippines, Tanzania, and Zimbabwe were classified either as controls (no Hg exposure from gold mining) or as having low (living in a gold-mining area) or high exposure (working as gold miners). U-Hg was analyzed by cold-vapor atomic absorption spectrometry. Eighteen SNPs in eight Hg-transporter genes were analyzed.

Results: U-Hg concentrations were higher among *ABCC2*/*MRP2* rs1885301 A–allele carriers than among GG homozygotes in all populations, though differences were not statistically significant in most cases. *MRP2* SNPs showed particularly strong associations with U-Hg in the subgroup with highest exposure (miners in Zimbabwe), whereas rs1885301 A–allele carriers had higher U-Hg than GG homozygotes [geometric mean (GM): 36.4 µg/g creatinine vs. 21.9; *p* = 0.027], rs2273697 GG homozygotes had higher U-Hg than A–allele carriers (GM: 37.4 vs. 16.7; *p* = 0.001), and rs717620 A–allele carriers had higher U-Hg than GG homozygotes (GM: 83 vs. 28; *p* = 0.084). The *SLC7A5*/*LAT1* rs33916661 GG genotype was associated with higher U-Hg in all populations (statistically significant for all Tanzanians combined). SNPs in *SLC22A6*/*OAT1* (rs4149170) and *SLC22A8*/*OAT3* (rs4149182) were associated with U-Hg mainly in the Tanzanian study groups.

Conclusions: SNPs in putative Hg-transporter genes may influence U-Hg concentrations.

Mercury (Hg) is a ubiquitous and highly reactive toxic metal. One of the most important sources of inorganic Hg exposure is gold mining because elemental Hg (Hg^0^) is widely used in gold extraction in small-scale gold mining (United Nations Environment Programme 2002). Hg^0^ is well absorbed in the lungs and has a high neurotoxic potential due to its ability to pass the blood–brain barrier (Nordberg et al. 2007). Hg^0^ is oxidized into mercuric Hg (Hg^2+^) in red blood cells and other tissues (Nordberg et al. 2007). Gold miners are at risk of Hg poisoning (Baeuml et al. 2011; Bose-O’Reilly et al. 2010a, 2010b; Drasch et al. 2001; Harari et al. 2012; Lettmeier et al. 2010), and Hg^0^ pollution from the gold-extraction process may also affect the health of other persons living close to mining areas (Bose-O’Reilly et al. 2010a, 2010b).

About 10–15 million persons work as gold miners, and approximately 30–50 million live in small-scale gold-mining areas (Spiegel et al. 2005). Earlier studies in the Philippines (Drasch et al. 2001), Indonesia (Bose-O’Reilly et al. 2010a), Tanzania (Bose-O’Reilly et al. 2010b), Zimbabwe (Lettmeier et al. 2010), and Ecuador (Harari et al. 2012) have shown that many persons who work or live in gold mining areas are highly exposed to inorganic Hg from gold mining, as can be seen from high concentrations of Hg in urine (U-Hg). However, large differences in U-Hg levels among persons with similar exposures have been reported.

Hg^2+^, formed from Hg^0^, is bound to glutathione and is excreted mainly in the urine. In contrast, there is little urinary excretion of methylmercury (MeHg), a Hg species for which exposure is mainly by consumption of fish. Hence, U-Hg is mainly a biomarker of exposure to inorganic Hg (i.e., Hg^0^ and Hg^2+^). Levels of U-Hg associated with detectable health effects in exposed workers fall between 10 and 30 μg/L (1 μg/L ≈ 1 µg/g creatinine) (Nordberg et al. 2007). U-Hg levels are lower in those who are not occupationally exposed to Hg. For example, persons in the Baltic region without amalgam fillings but with normal fish consumption had U-Hg levels < 1 μg/L. Although those who had dental amalgams had a few micrograms per liter of U-Hg, concentrations up to 50 μg/L have been reported for persons with many fillings (Skerfving et al. 1999).

As with other metals (Nordberg et al. 2007), genetics probably influences Hg uptake, distribution, and excretion. The few genetic variants that have been associated with Hg toxicokinetics identified to date have mainly been in glutathione-related genes (Custodio et al. 2005; Harari et al. 2012; Schlawicke Engström et al. 2008).

The different forms of Hg are thought to be transferred across cell membranes—either into or out of cells—by transporter proteins. The capacity of specific proteins for Hg transport has not been extensively studied and no specific Hg transporters have been identified. However, Hg forms complexes with small molecules such as amino acids that can mimic essential molecules recognized by transporter proteins. Some multispecific transporter families, such as organic anion transporters (OATs), system l-amino acid transporters (LATs), and ATP-binding cassette transporters (ABC transporters) are thought to alter Hg toxicokinetics (Bridges and Zalups 2005).

OATs are multispecific transport proteins located on the basolateral membrane. They mediate the uptake of a variety of substrates from renal blood. In rats, OAT1 and OAT3 have been shown to be responsible for > 60% of the renal tubular uptake of Hg^2+^ from renal blood into proximal tubule epithelial cells (Bridges and Zalups 2005).

LATs transport amino acids into cells in exchange for other amino acids. LAT1 and LAT2 can transport cysteine-bound MeHg, a complex that mimics certain amino acids (Bridges and Zalups 2005). LATs require interaction with SLC3A2 to function as transporters (del Amo et al. 2008), but nothing is known about their involvement in inorganic Hg transport.

ABC transporters participate in the transport of numerous toxicants across membranes, including anticancer agents and metals, and are major candidate genes for Hg transport. ABCC1, ABCC2, and ABCB1 (also known as multidrug resistance-associated proteins: MRP1, MRP2, and MDR1), are the most well-characterized ABC transporters. Up-regulation of ABC transporters by Hg^2+^ has been demonstrated in canine kidney cells (Aleo et al. 2005). ABCC2/MRP2 has been shown to mediate excretion of Hg^2+^ from proximal tubular cells into urine in rats (Bridges et al. 2011).

The aim of the present study was to estimate associations between single nucleotide polymorphisms (SNPs) in potential Hg-transporter genes and U-Hg as an indication of possible effects of the SNPs on the toxicokinetics of inorganic Hg.

## Materials and Methods

*Study subjects.* Participants included in the present analysis (*n* = 1,017) were gold miners, other persons living in gold-mining areas, or control individuals living in areas with no gold mining, including men and women > 12 years of age from the Philippines (*n* = 245) (Drasch et al. 2001), Indonesia (*n* = 330) (Bose-O’Reilly et al. 2010a), Tanzania (*n* = 226) (Bose-O’Reilly et al. 2010b), or Zimbabwe (*n* = 216) (Lettmeier et al. 2010) who were examined during 2002–2007 as a part of the United Nations Industrial Development Organization’s (UNIDO’s) Global Mercury Project. Former gold miners and one of two Indonesian study groups from the original study were excluded because of potential misclassification of Hg exposure (Bose-O’Reilly et al. 2010a) (those excluded were not part of the 1,017 participants). Only 1 participant had amalgam fillings, as determined by visual inspection. Spot urine samples were collected and acidified. Blood for DNA extraction was collected in EDTA tubes.

All participants were volunteers who provided written informed consent. The health examinations were performed in close cooperation with UNIDO and the United Nations Development Programme, as well as the regional health authorities and national ministries of health. All conditions were agreed upon between the governments and UNIDO, including extensive legal, formal, and ethical considerations. The conditions were approved by the governments and complied with all relevant national, state, and local regulations.

*Categories and biomarkers of Hg exposure*. Based on a questionnaire, the participants were divided into three exposure subgroups: *a*) controls (having no particular Hg exposure from gold mining, *n* = 139), *b*) low-exposure persons (living in Hg-contaminated mining areas, *n* = 297), and *c*) high-exposure persons (gold miners working with Hg^0^, *n* = 581). Participants in control subgroups were living in areas without any gold mining. Participants in the low-exposure subgroups were living in gold mining areas and were exposed to the Hg-contaminated environment. Participants in the high-exposure subgroups were living in the same areas as the low-exposure participants but were working as miners with immediate contact with Hg such as panning the crushed ore together with liquid Hg or smelting the amalgams. Six of the 1,017 study participants did not have data on U-Hg.

All U-Hg analyses were performed in the Institute of Forensic Medicine (LMU; Munich, Germany). Urine was analyzed for total Hg by cold-vapor atomic absorption spectrometry using a PerkinElmer 1100 B spectrometer) after capturing the Hg on a gold amalgam net, with a MHS 20 attachment (PerkinElmer, Norwalk, CT, USA). Sodium borohydride was applied for reducing Hg. The detection limit was 0.20 μg/L. For results below the limit of detection, the value was set to half the detection limit. U-Hg was adjusted for creatinine, analyzed by the Jaffe method (Mazzachi et al. 2000). Strict internal and external quality control was ensured. Quality controls with reference urine samples (ClinCal® urine (41 μg Hg/L) and ClinCeck® urine level I (3.5 μg Hg/L) and II (40 μg Hg/L) for trace elements (RECIPE Chemicals + Instruments GmbH, Munich, Germany) were continuously performed to ensure the accuracy of the results. The laboratory has demonstrated good performance in external quality control tests for Hg in human specimens. The laboratory has participated in round robin tests by the German DGKL (Deutsche Vereinte Gesellschaft für klinische Chemie und Laboratoriumsmedizin e.V., Bonn, Germany) for Hg in biological samples since 1998.

*Genotyping*. DNA was extracted from whole blood using the Qiagen DNA Blood Mini kit (Qiagen, Hilden, Germany). We selected SNPs based on three different criteria: *a*) functional impact according to the literature; *b*) potential functional impact according to position and type of SNP (specifically, non-synonymous SNPs that might affect the protein structure/enzyme activity or 5´ SNPs at putative promoter sites that can affect gene expression; National Center for Biotechnology Information 2006); and *c*) tagSNPs that capture as much of the genetic variation within a gene segment as possible because of linkage disequilibrium (LD) with other SNPs. TagSNPs were selected according to HapMap data (Thorisson et al. 2005) for YRI (Yoruba in Ibadan, Nigeria) as a proxy for the African populations and CHB (Han Chinese in Beijing, China) and JPT (Japanese in Tokyo, Japan) for Asians. In total, 18 SNPs were analyzed in genes coding for eight different transporters (Table 1). Genotyping was done using the iPLEX® Gold assay on the MassARRAY platform (Sequenom™, San Diego, CA, USA) for 15 SNPs. Additionally, three SNPs in *MRP2* (rs717620, rs2273697, and rs3740066) were analyzed by TaqMan allelic discrimination assay on an ABI 7900 instrument (Applied Biosystems, Foster City, CA, USA), according to the manufacturer’s instructions. Genotyping failed for 2 SNPs (*MRP1* rs41395947 and *SLC3A2* rs2282477). For the other SNPs, Sequenom™ genotyping failed for ≤ 2% and TaqMan genotyping failed for ≤ 4% of study participants. Five percent of the samples were reanalyzed for quality control purposes. There was perfect agreement between original and repeat genotyping runs for all SNPs.

**Table 1:** Characteristics of the SNPs^a^ under study in different countries.

Gene	ID of SNP^b^	Type of SNP^a^	Allele frequencies^a^						
			Indonesia	Philippines	Tanzania	Zimbabwe
MDR1 (ABCB1)	rs1045642	Synonymous, C > A > T	63/0/37	64/0/36	88/0/12	85/0/15
MDR1	rs2032582	Non-synonymous, Ala > Thr > Ser	52/5/43	43/9/48	98/0/2	100/0/0
MRP1 (ABCC1)	rs11075290	Intronic, C > T	57/43	69/31	71/29	74/26
MRP1	rs41395947	Non-synonymous, Cys > Ser	c	c	c	c
MRP2 (ABCC2)	rs1885301	5´ UTR/near gene, G > A	76/24	78/22	63/37	66/34
MRP2	rs717620	5´ UTR/near gene, G > A	80/20	85/15	99/1	99/1
MRP2	rs2273697	Non-synonymous, Val > Ile, G > A	97/3	89/11	79/21	83/17
MRP2	rs3740066	Synonymous, G > A	79/21	79/21	82/18	85/15
OAT1 (SLC22A6)	rs4149170	5´ UTR, G > A	80/20	88/12	62/38	53/47
OAT1	rs11568626	Non-synonymous, Pro > His > Arg > Leu	100/0/0/0	100/0/0/0	91/0/0/9	90/0/0/10
OAT3 (SLC22A8)	rs4149182	Intronic, G > C	76/24	79/21	63/37	62/38
OAT3	rs45566039	Non-synonymous, Arg > Ser	100/0c	100/0c	100/0c	100/0c
SLC3A2	rs2282477	3´ UTR, C > T	c	c	c	c
SLC3A2	rs77030286	5´ near gene, C > A	100/0	100/0	100/0	100/0
LAT1 (SLC7A5)	rs3815559	Intronic, G > C	89/11	90/10	65/35	72/28
LAT1	rs33916661	5´ UTR/near gene, A > G	79/21	82/18	67/33	65/35
LAT2	rs12879346	5´ UTR/near gene, A > T	64/36	66/34	63/37	67/33
LAT2	rs12588118	Intronic, G > C	89/11	87/13	84/16	82/18
UTR, untranslated region. aThe most common allele in all four populations is given first. bRs (reference SNP ID) numbers are from the National Center for Biotechnology Information (2006). cData are missing due to genotyping failure.												

*Bioinformatics.* Potential functional effects of SNPs with at least one statistically significant association (*p* < 0.05) with U-Hg in at least one study group were evaluated *in silico*. Promoter sites and potential transcription factor binding sites were analyzed by Genomatix software suite (Genomatix Software GmbH, Munich, Germany). This was evaluated for SNPs situated in the 5´ UTR (untranslated region) or introns.

*Statistical analysis.* In each country, deviations from Hardy-Weinberg equilibrium were tested using chi-square analysis, and LD was evaluated using Haploview (Barrett et al. 2005).

Natural log (ln) transformed U-Hg was the dependent variable. The general linear model procedure was used, in which genotypes with three levels can be modeled as nominal variables without assuming additive effects.

First, we estimated genetic associations with U-Hg for all persons in a country with adjustment for exposure subgroup (controls, low or high exposure) as categorical (nominal) variables, and after stratification by low or high exposure subgroup. In addition, we estimated associations adjusted for exposure subgroup according to continent (Africa, including Tanzania and Zimbabwe; or Asia, including Indonesia and the Philippines). We refer to these models as Model 1. Numbers of participants in the control groups were too small for stratum-specific analyses. Genotypes were modeled as categorical (nominal variables) coded 0, 1, or 2 according to the number of variant alleles, or were dichotomized with the common homozygous genotype. The genotype denoted last in the text (and in the first column in the tables) was used as reference (the common homozygote or the combination of the heterozygote and the common homozygote). However, in order to facilitate the interpretation of the results, other genotypes were sometimes used as reference, based on effect estimates.

Next, we estimated associations between genotypes (categorical, as above) and U-Hg by country or continent with adjustment for age (continuous) and sex, in addition to adjusting for exposure subgroup (categorical) or stratification according to low or high exposure (Model 2). In the analyses stratified for exposure group, adjustments were also made for Hg storage (categorical, categorized as never, at work, or at home). We did not include Hg storage in the analyses by country or continent because of strong colinearity with exposure subgroup (Pearson product-moment correlation coefficient, *r* = 0.8). Alcohol intake (never, > once a month, > once a week, or > once a day based on their response to the question “do you drink alcohol?” or categorized as “yes” or “no” for the Philippines) and body mass index [BMI (weight in kilograms divided by height in meters squared); continuous] were not included in models because they were correlated with sex (*r* around 0.5).

Statistical analyses were performed using SPSS (version 18; SPSS, Chicago, IL, USA). Statistical significance denotes *p* < 0.05 (two-tailed).

## Results

*Background data.* The study groups with the highest and lowest GM creatinine-adjusted U-Hg concentrations were from Zimbabwe and Tanzania, respectively (Table 2). Correlations (*r*) between U-Hg and potentially influential variables in the different populations are shown in Supplemental Material, Table S1 (http://dx.doi.org/10.1289/ehp.1204951). U-Hg was significantly correlated (all *p* < 0.001) with exposure subgroup (*r* = 0.46–0.85), Hg storage (*r* = 0.33–0.72), and male sex (*r* = 0.22–0.37) in all four countries, but was only weakly correlated with age (*r* = –0.12–0.093, *p* > 0.05). There was a positive correlation between U-Hg and alcohol consumption (*r* = 0.14–0.33, *p*-values of < 0.050; data not available for the Philippines) and positive correlations between alcohol consumption and other factors such as sex and exposure group (*r* = 0.3–0.5).

**Table 2:** Characteristics of the study populations.

Characteristic	Indonesia			Philippines			Tanzania			Zimbabwe		
	n	Value	n	Value	n	Value	n	Value
Age (years)a	330	33 (13–58)	245	37 (23–51)	226	33 (16–51)	216	28 (14–46)
Sex	330	245	226	215
Percent female	46	38	38	35
Storage of Hg (%)	325	243	224	215
Never	35	60	36	30
At home	39	24	42	63
At work	25	16	21	7
BMI (kg/m2)b	310	23 (18–31)	245	24 (19–30)	223	22 (17–31)	211	21 (16–26)
Alcohol consumption	329	240	226	216
Percent no	86	35	74	65
U-Hg (µg/g creatinine)b
All	330	4.2 (0.047–95)	244	2.9 (0.40–44)	221	1.0 (0.09–15)	216	7.7 (0.91–160)
Controls	21	0.38 (0.094–1.3)	40	2.0 (0.66–8.5)	32	0.17 (0.047–0.63)	45	0.12 (0.030–2.1)
Low exposure	106	2.8 (0.60–15)	113	1.3 (0.28–8.9)	52	0.41 (0.082–1.8)	24	5.3 (0.43–65)
High exposure	203	6.7 (0.91–160)	91	9.3 (1.3–61)	137	2.0 (0.20–20)	147	29 (2.3–210)
aMean (5th–95th percentile).bGeometric mean (5th–95th percentile).																

Genotyping was successful for 16/18 SNPs (Table 1). The minor alleles were the same in each country, for all SNPs (calculated for the total study population from each country, including the controls) except for *MDR1* rs2032582 in the Philippines. There were few differences in allelic frequencies among the different exposure subgroups within each country (Fisher’s exact test, not in table). In Indonesia, the allele frequency for the control group (*n* = 19) deviated from the low exposed for one SNP (rs717620). All SNPs were in Hardy-Weinberg equilibrium in all countries, except for *MRP2* rs717620 in Indonesia (χ^2^ = 7.1, *p* < 0.05) and Philippines (χ^2^ = 23, *p* < 0.05).

SNPs that were statistically significantly associated with U-Hg (*MRP2*, *OAT1*, *OAT3* and *LAT1*) in at least one country/subgroup are presented. For the other SNPs evaluated, no statistically significant associations with U-Hg were found.

Unstandardized β-coefficients derived from the comparison with the reference genotype (i.e., the estimated difference in ln-transformed U-Hg for two genotypes) and corresponding *p*-values and SEs are presented (Table 3 for *MRP2* and Table 4 for *LAT1*, *OAT1*, and *OAT3*). *p*-Value for the genotype term in the model are presented in the tables as well as when specifically stated in the text. All above are derived from Model 2 (adjusted model) if not stated otherwise.

**Table 3:** Multivariate analyses for *MRP2* SNPs on U-Hg concentrations (µg/g creatinine).

Location/ exposure subgroup	rs1885301				rs1885301			rs717620				rs717620			rs2273697		
	GGa	GA	AA	p-Value	GGa	AA + AG	p-Value	GGa	GA	AA	p-Value	GGa	AA + GA	p-Value	GGa	AA + GAb	p-Value
Indonesia
Low	p (n)	54	0.065 (47)	0.15 (5)	0.10	54	0.039 (52)	0.039	61	0.31 (42)	0.18 (8)	0.32	61	0.18 (42)	0.18	102	(2)	c
β (SE)	0.42 (0.23)	0.76 (0.52)	0.46 (0.22)	0.26 (0.25)	0.58 (0.44)	0.32 (0.24)
High	p (n)	119	0.14 (65)	0.42 (15)	0.29	119	0.12 (80)	0.12	135	0.11 (54)	0.10 (12)	0.046	135	0.40 (66)	0.40	184	0.22 (17)	0.21
β (SE)	0.34 (0.23)	0.32 (0.39)	0.34 (0.21)	0.39 (0.24)	–0.76 (0.46)	0.19 (0.23)	0.46 (0.38)
All	p (n)	190	0.075 (115)	0.19 (20)	0.12	190	0.046 (135)	0.046	213	0.11 (89)	0.52 (21)	0.18	213	0.25 (110)	0.25	306	0.20 (19)	0.20
β (SE)	0.28 (0.16)	0.40 (0.31)	0.30 (0.15)	0.27 (0.17)	–0.20 (0.31)	0.18 (0.16)	0.41 (0.32)
Philippines	
Low	p (n)	71	0.11 (34)	0.59 (7)	0.20	71	0.23 (41)	0.23	76	0.13 (23)	d	0.13	76	0.13 (23)	0.13	82	0.76 (19)	0.76
β (SE)	0.33 (0.20)	–0.20 (0.38)	0.23 (0.19)	0.39 (0.25)	0.37 (0.24)	0.08 (0.25)
High	p (n)	55	0.70 (29)	0.24 (7)	0.49	55	0.45 (36)	0.45	51	0.95 (22)	0.51 (5)	0.45	51	0.86 (27)	0.86	56	0.56 (22)	0.55
β (SE)	0.11 (0.29)	0.61 (0.51)	0.21 (0.27)	–0.02 (0.33)	0.40 (0.62)	0.05 (0.31)	–0.19 (0.33)
All	p (n)	148	0.15 (78)	0.33 (16)	0.27	148	0.11 (94)	0.11	158	0.061 (49)	0.30 (9)	0.12	158	0.041 (58)	0.041	172	0.45 (46)	0.44
β (SE)	0.21 (0.15)	0.27 (0.28)	0.22 (0.14)	0.33 (0.17)	0.38 (0.36)	0.33 (0.16)	–0.13 (0.18)
Tanzania	
Low	p (n)	16	0.49 (27)	0.58 (7)	0.75	16	0.44 (34)	0.44	53	(0)	(0)	c	53	(0)	c	27	0.61 (24)	0.61
β (SE)	0.21 (0.30)	0.24 (0.43)	0.22 (0.28)	0.14 (0.28)
High	p (n)	54	0.62 (63)	0.93 (19)	0.88	54	0.66 (82)	0.66	131	(2)	(1)	c	131	(3)	c	85	0.075 (46)	0.075
β (SE)	0.13 (0.26)	0.03 (0.38)	0.11 (0.25)	–0.45 (0.25)
All	p (n)	85	0.55 (101)	0.92 (32)	0.82	85	0.60 (133)	0.60	214	0.93 (5)	d	0.93	214	0.93 (5)	0.93	134	0.40 (80)	0.40
β (SE)	0.11 (0.19)	0.03 (0.26)	0.09 (0.17)	–0.05 (0.57)	–0.05 (0.57)	–0.15 (0.18)
Zimbabwe	
Low	p (n)	7	0.63 (17)	0.63	7	0.63 (17)	0.63	24	(0)	(0)	c	24	(0)	c	17	0.97 (7)	0.97
β (SE)	0.32 (0.65)	0.32 (0.65)	0.02 (0.64)
High	p (n)	63	0.036 (65)	0.033 (18)	0.030	63	0.013 (83)	0.013	136	0.19 (5)	d	136	0.19 (5)	95	0.001 (46)	0.001
β (SE)	0.47 (0.23)	0.71 (0.33)	0.53 (0.21)	0.77 (0.58)	0.19	0.77 (0.58)	0.19	–0.78 (0.22)
All	p (n)	90	0.076 (103)	0.15 (22)	0.14	90	0.049 (125)	0.049	204	0.17 (6)	d	204	0.17 (6)	147	0.022 (63)	0.022
β (SE)	0.33 (0.18)	0.43 (0.30)	0.35 (0.18)	0.72 (0.53)	0.17	0.17	0.72 (0.53)	0.17	–0.44 (0.19)
Asia	p (n)	339	0.004 (194)	0.13 (36)	0.009	339	0.002 (230)	0.002	373	0.004 (138)	0.92 (30)	0.015	373	0.010 (168)	0.010	478	0.78 (65)	0.78
	β (SE)	0.33 (0.11)	0.34 (0.22)	0.33 (0.11)	0.38 (0.13)	0.03 (0.25)	0.31 (0.12)	0.05 (0.17)
Africa	p (n)	177	0.24 (206)	0.86 (55)	0.49	177	0.30 (261)	0.30	418	0.26 (11)	d	0.15	418	0.26 (11)	0.26	281	0.025 (143)	0.025
	β (SE)	0.21 (0.18)	0.05 (0.27)	0.17 (0.17)	0.59 (0.53)	0.26	0.59 (0.53)	–0.40 (0.18)
Multivariate analyses were adjusted for age, sex, and Hg storage in the “low” and “high” groups, and for age, sex, and exposure subgroup in the “All,” “Asia,” and “Africa” groups. Genotype, sex, exposure subgroup, and Hg storage were modeled as categorical (nominal) variables, and age was modeled as a continuous variable. U-HG was ln transformed. Unstandardized β-coefficients were derived from the comparison with the reference genotype, i.e., the estimated difference in ln-transformed U-Hg for two genotypes, as well as p-values and SE for these comparisons. p-Value is for the genotype term in the model. Control subgroups are not shown separately, but are included in the “All,” “Asia,” and “Africa” groups. aReference genotype. bRs2273697 AA + AG genotypes were pooled in all populations because of a low number of individuals with AA genotype. cNo analyses were run due to a low number (≤ 3) of individuals for all genotypes but one. dThe genotype was pooled with the heterozygotes due to a low number (≤ 3) of individuals.																						

**Table 4:** Multivariate analyses for *LAT1*, *OAT1*, and *OAT3* SNPs on U-Hg concentrations (µg/g creatinine).

Location/exposure subgroup	LAT1 rs33916661							OAT1 rs4149170							OAT3 rs4149182						
	GG^a^	GA	AA	p-Value	GG^a^	GA	AA	p-Value	GG^a^	CG	CC	p-Value
Indonesia
Low	p (n)	6	0.41 (40)	0.61 (60)	0.63	64	0.10 (35)	0.031 (7)	0.050	56	0.15 (42)	0.97 (8)	0.33
β (SE)	–0.41 (0.50)	–0.24 (0.48)	–0.39 (0.24)	–0.97 (0.45)	0.34 (0.24)	–0.02 (0.43)
High	p (n)	10	0.68 (61)	0.67 (125)	0.20	135	0.68 (57)	0.98 (9)	0.92	125	0.60 (64)	0.19 (10)	0.33
β (SE)	–0.20 (0.49)	0.20 (0.48)	–0.10 (0.23)	–0.01 (0.50)	–0.12 (0.22)	0.62 (0.48)
All	p (n)	16	0.37 (106)	0.91 (199)	0.19	215	0.31 (93)	0.40 (18)	0.46	190	0.77 (116)	0.41 (19)	0.70
β (SE)	–0.32 (0.35)	–0.04 (0.34)	–0.17 (0.16)	–0.27 (0.32)	0.05 (0.16)	0.26 (0.32)
Philippines
Low	p (n)	0	(33)	(80)	b	92	0.60 (18)	c	0.60	76	0.17 (30)	0.29 (6)	0.18
β (SE)	–0.086 (0.16)	–0.28 (0.20)	0.44 (0.41)
High	p (n)	4	0.84 (30)	0.77 (53)	0.94	65	0.72 (25)	c	0.72	52	0.050 (35)	0.63 (4)	0.14
β (SE)	–0.13 (0.66)	–0.19 (0.64)	–0.11 (0.30)	–0.54 (0.27)	–0.31 (0.65)
All	p (n)	4	0.80 (77)	0.74 (158)	0.91	186	0.72 (49)	0.47 (4)	0.73	150	0.028 (81)	0.93 (11)	0.081
β (SE)	–0.14 (0.55)	–0.18 (0.54)	–0.06 (0.17)	–0.39 (0.54)	–0.32 (0.15)	0.03 (0.33)
Tanzania
Low	p (n)	5	0.57 (19)	0.76 (28)	0.31	12	0.010 (24)	0.022 (16)	0.025	11	0.004 (23)	0.002 (17)	0.004
β (SE)	–0.28 (0.48)	0.15 (0.49)	–0.81 (0.30)	–0.79 (0.33)	–0.91 (0.30)	–1.05 (0.32)
High	p (n)	18	0.060 (59)	0.22 (58)	0.16	48	0.49 (64)	0.039 (25)	0.024	46	0.85 (66)	0.046 (25)	0.059
β (SE)	–0.71 (0.37)	–0.46 (0.37)	0.18 (0.27)	–0.70 (0.34)	0.05 (0.27)	–0.69 (0.34)
All	p (n)	26	0.004 (93)	0.064 (100)	0.015	72	0.79 (102)	0.024 (47)	0.023	67	0.81 (105)	0.010 (48)	0.017
β (SE)	–0.79 (0.28)	–0.51 (0.27)	0.05 (0.19)	–0.54 (0.24)	–0.05 (0.19)	–0.62 (0.24)
Zimbabwe
Low	p (n)	3	(7)	(13)	b	6	0.20 (14)	0.27 (4)	0.074	6	0.46 (14)	0.19 (4)	0.13
β (SE)	0.83 (0.62)	–0.84 (0.73)	0.49 (0.64)	–1.04 (0.76)
High	p (n)	25	0.90 (62)	0.51 (58)	0.71	44	0.59 (65)	0.81 (37)	0.72	43	0.60 (64)	0.70 (39)	0.64
β (SE)	–0.04 (0.30)	–0.20 (0.30)	0.13 (0.25)	–0.07 (0.28)	0.13 (0.25)	–0.11 (0.28)
All	p (n)	31	0.61 (87)	0.37 (94)	0.65	64	0.57 (100)	0.28 (51)	0.22	63	0.72 (99)	0.20 (53)	0.24
β (SE)	–0.14 (0.27)	–0.24 (0.26)	0.12 (0.20)	–0.26 (0.24)	0.07 (0.21)	–0.30 (0.24)
Asia	p (n)	20	0.25 (183)	0.32 (359)	0.51	203	0.88 (142)	0.32 (22)	0.6	341	0.48 (198)	0.48 (30)	0.55
β (SE)	–0.34 (0.30)	–0.29 (0.29)	–0.02 (0.12)	–0.28 (0.28)	–0.08 (0.11)	0.17 (0.24)
Africa	p (n)	57	0.017 (181)	0.024 (198)	0.046	136	0.34 (207)	0.28 (98)	0.12	230	0.66 (209)	0.20 (101)	0.19
β (SE)	–0.63 (0.26)	–0.59 (0.26)	0.18 (0.19)	–0.25 (0.23)	0.09 (0.19)	–0.29 (0.23)
Multivariate analyses were adjusted for age, sex, and Hg storage in the “low” and “high” groups, and for age, sex, and exposure subgroup in the “All,” “Asia,” and “Africa” groups. Genotype, sex, exposure subgroup, and Hg storage were modeled as categorical (nominal) variables, and age was modeled as a continuous variable. U-HG was natural ln transformed. Unstandardized β-coefficients were derived from the comparison with the reference genotype, i.e., the estimated difference in ln-transformed U-Hg for two genotypes, as well as p-values and SE for these comparisons. p-Value is for the genotype term in the model. eControl subgroups are not shown separately, but are included in the “All,” “Asia,” and “Africa” groups. aReference genotype. bDenotes that no analyses were run because no individuals had the reference genotype. cThe genotype was pooled with the heterozygotes due to a low number (≤ 3) of individuals.																										

*MRP2 and U-Hg*. The *MRP2* rs1885301 A–allele carriers had higher observed creatinine-adjusted GM U-Hg concentrations than those with the GG genotype in most populations (overall and according to high or low exposure within each country) [see Supplemental material, Table S2 (http://dx.doi.org/10.1289/ehp.1204951)]. When we pooled data from the Asian or African countries respectively, a significant association with U-Hg was seen for Asia (*p* = 0.002, β = 0.33, *SE* = 0.11, AA + AG vs. GG), but not for Africa (*p* = 0.30, β = 0.17, *SE* = 0.17, AA + AG vs. GG).

Bioinformatic analyses indicated that rs1885301 is situated 5´ of the promoter and that the G > A substitution results in loss of one transcription-factor binding site [Fetal Alz-50 clone 1 (FAC1)] and gain of another site (FAST-1 SMAD interacting protein).

*MRP2* rs717620 was rare in the African countries, where frequencies of the A allele were only 1%, as compared to 15–20% in Asia. Persons with one or two copies of the A allele had higher U-Hg concentrations in all exposure subgroups in which at least four individuals carried this allele [see Supplemental Material, Table S2 (http://dx.doi.org/10.1289/ehp.1204951)]. This was statistically significant in the Philippines (*p* = 0.041, β = 0.33, *SE* = 0.16, AA vs. AG + GG). In the pooled analyses, associations were in the same directions: persons with one or two copies of the A allele had higher U-Hg concentrations (Asia: *p* = 0.010, β = 0.31, *SE* = 0.12, AA vs. AG + GG; Africa: *p* = 0.26, β = 0.59, *SE* = 0.53, AA vs. AG + GG). As noted above, this SNP was in Hardy-Weinberg disequilibrium in the Asian populations. *In silico* analysis indicated that rs717620 is situated in a promoter site, and that the G > A substitution leads to loss of two transcription-factor binding sites (LEF-1 and SOX4).

The non-synonymous *MRP2* rs2273697 was significantly associated with U-Hg among high-exposed miners in Zimbabwe only, such that carriers of the A allele (Ile) had lower U-Hg concentrations than persons with no copies of the A allele (*p* = 0.001, β = –0.78, *SE* = 0.22, AA + AG vs. GG). The A allele was also associated with lower U-Hg among high-exposed miners in the Philippines (who had the second-highest U-Hg among all population subgroups), although this association was not statistically significant (*p* = 0.55, β = –0.19, *SE* = 0.33, AA + AG vs. GG), as well as among all African participants combined (*p* = 0.025, β = –0.40, *SE* = 0.18, AA + AG vs. GG). However, the association was in the opposite direction in Indonesia (all persons), although not statistically significant (*p* = 0.20, β = 0.41, *SE* = 0.32, AA + AG vs. GG).

Overall, associations with the three *MRP2* SNPs discussed above were particularly strong in the highest exposed subgroup, gold miners in Zimbabwe. In this subgroup, rs1885301 A–allele carriers had higher U-Hg than those with the GG genotype (observed GM: 36.4 vs. 21.9 µg/g creatinine, Model 1 unadjusted *p* = 0.027, β = 0.51, *SE* = 0.23, AA + AG vs. GG), rs2273697 GG genotype had higher U-Hg concentrations than A–allele carriers (observed GM: 37.4 vs. 16.7 µg/g creatinine, Model 1 unadjusted *p* = 0.001, β = 0.81, *SE* = 0.24, AA + AG vs. GG), and rs717620 A–allele carriers had higher U-Hg concentrations than those with the GG genotype (observed GM 83.1 vs. 27.7 µg/g creatinine, Model 1 unadjusted *p* = 0.084, β = 1.1, *SE* = 0.63, AA + AG vs. GG).

Rs2273697 was not in LD with rs1885301 or rs717620 in any population. Rs717620 was in LD with rs1885301 (the common G alleles of both SNPs were linked to each other) in Indonesia (*R^2^* = 0.69) and the Philippines (*R^2^* = 0.40), but not in the African study groups (*R^2^* = 0.02 in both countries).

*LAT1 and U-Hg.* For *LAT1* rs33916661, persons with the rare GG genotype showed higher observed GM U-Hg concentrations than persons carrying GA or AA genotypes when all participants were combined by country or continent as well as in the high exposed subgroups [see Supplemental Material, Table S3 (http://dx.doi.org/10.1289/ehp.1204951)]. However, the associations were statistically significant only in Tanzania (Table 4, *p* = 0.015 for the genotype term in the adjusted models, *p* = 0.064, β = –0.51, *SE* = 0.27 for the comparison of AA vs. GG and *p* = 0.004, β = –0.79, *SE* = 0.28 for AG vs. GG) and when the African countries were pooled (*p* = 0.046 for the genotype term, *p* = 0.024, β = –0.59, *SE* = 0.26 for AA vs. GG, *p* = 0.017, β = –0.63, *SE* = 0.26 for AG vs. GG). In Tanzania (all persons) and in the African group, the group with GG genotype had about twice the GM U-Hg concentration as those with AG or AA genotypes (see Supplemental Material, Table S3). This SNP is situated in the 5´ untranslated region, and *in silico* analysis indicated that it results in the loss of two transcription-factor binding sites: Paired box 5 B cell–specific activator protein new (PAX5), and Avian C-type LTR CCAAT box binding factors, Zinc finger protein 217 (ZNF217).

*OATs and U-Hg.* Two of three SNPs evaluated in OATs (*OAT1* rs4149170 and *OAT3* rs4149182) were associated with lower U-Hg in the African populations (Table 4). These genotypes were rare in the Asian populations (2–4%). In most cases, persons with the *OAT1* rs4149170 AA or the *OAT3* rs4149182 CC genotype had lower GM U-Hg concentrations than those with AG or GG and CG or GG genotypes, respectively [see Supplemental Material, Table S3 (http://dx.doi.org/10.1289/ehp.1204951)]. Statistically significant associations were seen in all groups in Tanzania: persons with AA or CC genotypes had lower U-Hg concentrations than the GG genotypes (reference). For rs4149170, *p*-values for the genotype term were 0.025 (low exposed), 0.024 (high exposed), and 0.023 (all persons). When comparing AA with GG: *p* = 0.022, β = –0.79, *SE* = 0.33 for low exposed, *p* = 0.039, β = –0.70, *SE* = 0.34 for high exposed, and *p* = 0.024, β = –0.54, *SE* = 0.24 for all persons. For rs4149182, *p*-values for the genotype term were 0.004 (low exposed), 0.059 (high exposed), and 0.017 (all persons). When comparing CC with GG: *p* = 0.002, β = –1.05, *SE* = 0.32 for low exposed, *p* = 0.046, β = –0.69, *SE* = 0.34 for high exposed, and *p* = 0.010, β = –0.62, *SE* = 0.24 for all persons. In these groups, the U-Hg concentrations were about 50% lower for persons with AA or CC compared to GG (see Supplemental Material, Table S3). Persons with AA or CC genotypes had lower U-Hg in Zimbabwe, although these associations did not reach statistical significance. No statistically significant associations were seen for these SNPs when the African or Asian study groups were pooled. *OAT1* rs4149170 and *OAT3* rs4149182 were in LD in the African populations (the common G alleles of both SNPs were linked to each other: Tanzania, *R^2^* = 0.82; Zimbabwe, *R^2^* = 0.55) but this was not found in the Asian populations (Indonesia, *R^2^* = 0.06; Philippines, *R^2^* = 0.01). *In silico* analyses indicate that rs4149182 in *OAT3* is situated in a binding site for metal-regulatory transcription factor 1 (MTF1), and that the C > G transversion results in loss of the MTF1-binding site.

Because there were correlations between alcohol consumption and sex, and BMI and sex, we did not include them in the models above in order to avoid over-adjustment. However, we conducted a sensitivity analysis and compared model estimates adjusted for either alcohol consumption or BMI instead of sex. Results were generally consistent with those adjusted for sex (data not shown). Because sex was correlated with U-Hg concentrations, we also compared associations between men and women by including an interaction term (gene × sex) in the models or by stratifying by sex in addition to the stratifications for country/continent and exposure group. However, no clear patterns could be seen (data not shown), probably due to a too low power.

## Discussion

In this study, we report evidence suggesting that variations in the transporter gene *MRP2* influence U-Hg in persons exposed to Hg^0^. Associations between *MRP2* rs1885301 and U-Hg were observed in study populations from two continents, and associations with two *MRP2* SNPs (rs1885301 and rs2273697) appeared to be particularly strong in gold miners in Zimbabwe, the study subgroup with highest exposure. Polymorphisms in *LAT1, OAT1*, and *OAT3* were associated with U-Hg, but statistically significant associations were mainly observed in Tanzania. *In silico* analyses indicated that several of the SNPs associated with U-Hg alter putative transcription-factor binding sites, which indicate that they may influence gene expression.

This study had several strengths. The exposed populations were fairly large, ranging between 171 and 309 persons in the different countries, although there were relatively few participants in the control populations that were not exposed to Hg^0^ used in gold mining. U-Hg determinations and genotyping were done by the same methods in the same laboratories, and with good quality control. U-Hg concentrations indicated a wide range of exposures (with GM in the different exposure groups ranging from 0.12 to 29 µg/g creatinine in Zimbabwe participants, for example.)

For some polymorphisms, however, the frequencies were low, which limited our ability to identify associations or consistent patterns across populations. We cannot exclude the possibility that differences in genetic background between exposure subgroups within each country might exist for other genes that influence Hg metabolism, since controls were not always living in exactly the same area, and gold miners were work migrants in many cases.

The allelic frequencies varied among the populations, as did the LD patterns (data not shown). Thus, we performed separate analyses for each country. We had a problem of multiple inference. However, the main candidate gene, well-founded in the literature, was *MRP2*, while associations with U-Hg for the other genes evaluated were plausible but less well supported. To increase our power to detect associations we merged the African and Asian populations in separate analyses. These results must be interpreted with caution due to differences in mining practices that may have influenced exposure levels, including differences in frequency and intensity of gold-amalgam burning, which is the main route of Hg^0^ exposure (Harari et al. 2012). The Asian countries were more similar in exposure and U-Hg concentrations than the African ones, and thus provided the strongest results in the merged analyses. There were no large variations in allele frequencies between the countries within the pooled Asian and African groups.

Hg excretion among gold miners varies over time, in association with the time since the last burning of gold amalgam (Harari et al. 2012), which could obscure genetic effects, if present. This may explain why associations with SNPs in *OAT1, OAT3* and *LAT1* mainly were seen in Tanzania, where the exposure was lowest, but was probably the most stable over time.

Alcohol intake may modify the levels of U-Hg. Alcohol consumption has been shown to increase the rate of exhalation of mercury and to reduce U-Hg levels (Clarkson and Magos 2006). However, we decided not to include it in the statistical models in order to avoid over-adjustment; alcohol intake was positively associated with both exposure group and sex. The positive correlation between alcohol consumption and U-Hg in our study populations was inconsistent with expectations, which makes us believe that the association was confounded by a positive association between alcohol consumption and exposure group/sex rather than being a true effect.

Sex differences in metal toxicokinetics and toxicodynamics have been described for some metals, including MeHg (Vahter et al. 2007). Sex was correlated with U-Hg in our study populations, but was also associated with other factors related to exposure (alcohol consumption, BMI, work tasks–men were more likely than women to report that they worked with smelting amalgam). We did not find any gene × sex interactions for U-Hg, but we had limited power for these analyses, especially in the high-exposed groups where women constituted 20–30% of the study participants.

Transporter proteins are involved in active uptake of compounds, and as the Hg uptake in the lung is through passive absorption, we believe it is unlikely that lung absorption of Hg^0^ is affected by transporter genes (Nordberg et al. 2007). Our hypothesis is that, given the same exposure, persons with higher U-Hg would have enhanced excretion.

MRP2 is an important regulator of metal toxicity in different species, such as in rats and *Arabidopsis thaliana* (mouse-ear cress) (Liu et al. 2001; Song et al. 2010). Hg induces up-regulation of *MRP2* and expression of MRP2 protein *in vitro* (Aleo et al. 2005). MRP2 transports Hg out of cells and MRP2 levels are high in the kidney (Wu et al. 2009). Increased MRP2 expression in the kidney may increase U-Hg excretion. Two of the *MRP2* SNPs that we evaluated (rs1885301 and rs717620) are situated 1,500 base pairs apart, both at the 5´ end of the gene, which is associated with regulation of gene expression. However, there are no data for the influence of the gene expression for rs1885301. For rs717620, the data are inconclusive; no effect of the variant A allele was found on gene or protein expression levels in liver cells or enterocytes in three studies analyzing expression (Deo et al. 2012; Moriya et al. 2002; Zhang et al 2010), whereas lower mRNA levels in kidney tissue were reported in one study (Haenisch et al. 2007). Since the *in silico* analysis showed a loss of two activating transcription factors for the rs717620 A allele, this finding is in line with the lower mRNA levels found in Haenisch et al. (2007). We did not identify any reports concerning effects of *MRP2* SNPs on metal transport.

Previously, LAT1 has only been shown to transport MeHg in *Xenopus laevis* oocytes (Simmons-Willis et al. 2002). *LAT1* rs33916661 is situated at the 5´ end of the gene. Little is known about the functional impact of this polymorphism. Our *in silico* analysis suggests that the GG genotype, which was associated with higher U-Hg concentrations in most population subgroups, results in the loss of two transcription-factor binding sites and a gain of one new site.

OAT1 and OAT3 mediate uptake of Hg^2+^ into cells, particularly in the kidney (Bridges and Zalups 2005). Rs4149182 was found to be situated in an MTF1-binding site, a so-called metal-responsive element. MTF1 induces a positive regulation of transcription in response to metal ions. The C > G transversion resulted in loss of an MTF1-binding site.

Further functional studies are, however, needed to prove causation between the SNPs identified and Hg metabolism. False-positive results may have arisen due to multiple testing of several genetic markers in different populations. Furthermore, the SNPs identified may not be functional, but may be linked to other unknown and truly functional SNPs. There is also a problem with misclassification of exposure subgroups, as the miners were placed in the same category according to their working circumstances and not based on true exposure levels.

## Conclusions

Several SNPs in genes coding for transporters were associated with U-Hg concentrations in at least some study populations. Results support the hypothesis that transporters may be involved in the toxicokinetics of Hg. In particular, some *MRP2* variants were associated with U-Hg in study groups from different countries.

## Supplemental Material

(94 KB) PDF
