# Supplementary material for: Polymorphisms in Genes Encoding Potential Mercury Transporters and Urine Mercury Concentrations in Populations Exposed to Mercury Vapor from Gold Mining
Source: Environ Health Perspect. 2012 Oct 9;121(1):85–91. doi: 10.1289/ehp.1204951 (PMC3553430; doi:10.1289/ehp.1204951)
Supplement: (94 KB) PDF [file ehp.1204951.s001.pdf]

Supplemental Material

# Polymorphisms in Genes Encoding Potential Mercury Transporters and Urine Mercury Concentrations in Populations Exposed to Mercury Vapor from Gold Mining

Karin Engström[1], Shegufta Ameer[1], Ludovic Bernaudat[2], Gustav Drasch[3], Jennifer Baeuml[4], Staffan Skerfving[1], Stephan Bose-O'Reilly[4,5] Karin Broberg[1]

[1] Department of Laboratory Medicine, Division of Occupational and Environmental Medicine, Lund University, Lund, Sweden

[2] United Nations Industrial Development Organization, Vienna, Austria

[3] Institute of Forensic Medicine, Ludwig-Maximilians-University, Munich, Germany

[4] Institute of Public Health, Medical Decision Making and Health Technology Assessment, UMIT – University for Health Sciences, Medical Informatics and Technology, Hall i.T., Austria

[5] Institute and Outpatient Clinic for Occupational, Social and Environmental Medicine, University Hospital Munich, Munich, Germany

## Table of contents

Table S1. Pearson correlations (*r*) between urinary mercury and potentially influential variables in the different study populations.

Table S2. Unadjusted geometric mean concentrations of mercury in urine (µg/g creatinine) and numbers of observations according to *MRP2* genotype by exposure subgroup, country and continent.

Table S3. Unadjusted geometric mean concentrations of mercury in urine (µg/g creatinine) according to *LAT1* rs33916661, *OAT1* 4149170 and *OAT3* 4149182 genotypes by exposure subgroup, country and continent.

Supplemental Material Table S1. Pearson correlations (*r*) between urinary mercury [a] and potentially influential variables in the different study populations.

| Country/ Continent | Correlation/ p-value | Exposure group | Hg storage | Age | Gender [b] |
|---|---|---|---|---|---|
| Indonesia | *r* | *0.46* | *0.33* | *0.093* | *0.22* |
| | p | <0.001 | <0.001 | 0.090 | <0.001 |
| Philippines | *r* | *0.51* | *0.53* | *-0.12* | *0.26* |
| | p | <0.001 | <0.001 | 0.055 | <0.001 |
| Tanzania | *r* | *0.61* | *0.52* | *-0.077* | *0.37* |
| | p | <0.001 | <0.001 | 0.25 | <0.001 |
| Zimbabwe | *r* | *0.85* | *0.72* | *-0.015* | *0.33* |
| | p | <0.001 | <0.001 | 0.82 | <0.001 |
| Asia | *r* | *0.50* | *0.42* | *-0.001* | *0.22* |
| | p | <0.001 | <0.001 | 0.99 | <0.001 |
| Africa | *r* | *0.67* | *0.63* | *-0.13* | *0.31* |
| | p | <0.001 | <0.001 | 0.008 | <0.001 |

[a]Creatinine-adjusted, natural log- (ln-) transformed.

[b] Female=0, male=1.

Supplemental Material Table S2. Unadjusted geometric mean concentrations of urinary mercury (µg/g creatinine) and numbers of observations according to *MRP2* genotype by exposure subgroup[a], country and continent.

| Country/ Continent | Indonesia | | | Philippines | | | Tanzania | | | Zimbabwe | | | Asia | Africa |
|---|---|---|---|---|---|---|---|---|---|---|---|---|---|---|
| Exposure subgroup | Low | High | All | Low | High | All | Low | High | All | Low | High | All | All | All |
| *Genotype* | | | | | | | | | | | | | | |
| **Rs1885301** | | | | | | | | | | | | | | |
| *AA* | 4.9 (5) | 7.1 (15) | 6.4 (20) | 0.94 (7) | 16.5 (7) | 3.9 (16) | 0.47 (7) | 2.2 (19) | 0.92 (32) | [b] | 43.0 (18) | 16.5 (22) | 5.1 (36) | 3.0 (55) |
| *GA* | 3.4 (47) | 7.3 (65) | 4.9 (115) | 1.8 (34) | 9.8 (29) | 3.4 (78) | 0.42 (27) | 2.1 (63) | 1.1 (101) | 5.6 (17) | 34.8 (65) | 7.8 (103) | 4.2 (194) | 2.9 (206) |
| *GG* | 2.2 (54) | 6.2 (119) | 3.6 (190) | 1.2 (71) | 8.4 (55) | 2.6 (148) | 0.37 (16) | 1.8 (54) | 0.89 (85) | 4.6 (7) | 21.9 (63) | 6.1 (90) | 3.1 (339) | 2.4 (177) |
| *AA+AG* | 3.5 (52) | 7.2 (80) | 5.1 (135) | 1.6 (41) | 10.8 (36) | 3.4 (94) | 0.37 (34) | 2.2 (82) | 1.0 (133) | 5.6 (17) | 36.4 (83) | 8.9 (125) | 4.4 (230) | 2.9 (261) |
| *GG* | 2.2 (54) | 6.2 (119) | 3.6 (190) | 1.2 (71) | 8.4 (55) | 2.6 (148) | 0.43 (16) | 1.8 (54) | 0.89 (85) | 4.6 (7) | 21.9 (63) | 6.1 (90) | 3.1 (339) | 2.4 (177) |
| **Rs717620** | | | | | | | | | | | | | | |
| *AA* | 4.5(8) | 2.5 (12) | 2.9 (21) | [b] | 16.2 (5) | 5.8(9) | (0) | (1)[c] | [b] | (0) | (0) | (0) | 3.6 (30) | [b] |
| *GA* | 3.1(42) | 8.8 (54) | 5.6 (89) | 1.9 (23) | 9.5 (22) | 4.3 (49) | (0) | (2)[c] | 2.0 (5) | (0) | 83 (5) | 24.9 (6) | 5.1 (138) | 4.8 (11) |
| *GG* | 2.4(61) | 6.5 (135) | 3.8 (213) | 1.1 (76) | 9.7 (51) | 2.5 (158) | (53)[c] | (131)[c] | 2.0 (214) | (24)[c] | 28 (136) | 7.0 (204) | 3.2 (373) | 2.6 (418) |
| *AA+GA* | 3.3(42) | 7.0 (66) | 5.0 (110) | 1.9 (23) | 10.5 (27) | 4.5 (58) | (0) | (3)[c] | 2.0 (5) | (0) | 83 (5) | 24.9 (6) | 4.8 (168) | 4.8 (11) |
| *GG* | 2.4 (61) | 6.5 (135) | 3.8 (213) | 1.1 (76) | 9.7 (51) | 2.5 (158) | (53)[c] | (131)[c] | 2.0(214) | (24)[c] | 28 (136) | 7.0 (204) | 3.2 (373) | 2.6 (418) |
| **Rs2273697** | | | | | | | | | | | | | | |
| *AA+GA*[d] | (2)[c] | 11.2 (17) | 9.2 (19) | 1.3 (19) | 8.3 (22) | 3.2 (46) | 0.50 (24) | 1.6 (46) | 0.85 (80) | 5.4 (7) | 16.7 (46) | 7.3 (63) | 3.5 (65) | 2.2 (143) |
| *GG* | 2.8(102)[c] | 6.3 (184) | 4.0 (306) | 1.3 (82) | 10.7 (56) | 2.8 (172) | 0.36 (27) | 2.4 (85) | 1.0 (134) | 5.3 (17) | 37.4 (95) | 7.3 (147) | 4.4 (478) | 2.9 (281) |

[Table S2 continued]

[a]Control subgroups are not shown separately, but are included in the “All” group.

[b]Denotes that the genotype is pooled with heterozygotes, due to a low number of individuals (3 or less).

[c]Data is not shown, due to a low number of individuals (3 or less) for all genotypes but one.

[d]Rs2273697 AA+AG genotypes were pooled in all populations due to a low number of individuals with AA genotype.

Supplemental Material Table S3.Unadjusted geometric mean concentrations of urinary mercury (µg/g creatinine)[a] according to *LAT1* rs33916661, *OAT1* 4149170 and *OAT3* 4149182 genotypes by exposure subgroup[b], country and continent.

| Country/ Continent | Indonesia | | | Philippines | | | Tanzania | | | Zimbabwe | | | Asia | Africa |
|---|---|---|---|---|---|---|---|---|---|---|---|---|---|---|
| Exposure subgroup | Low | High | All | Low | High | All | Low | High | All | Low | High | All | All | All |
| *Genotype* | | | | | | | | | | | | | | |
| ***LAT1* Rs33916661** | | | | | | | | | | | | | | |
| *GG* | 3.4 (6) | 8.1 (10) | 5.8 (16) | (0) | 11.2 (4) | 11.2 (4) | 0.45 (5) | 3.3 (18) | 1.8 (26) | [c] | 35.1 (25) | 17.4 (31) | 6.6 (20) | 6.2 (57) |
| *GA* | 2.5 (40) | 5.3 (61) | 3.6 (106) | 1.3 (33) | 10.2 (30) | 3.2 (177) | 0.35 (19) | 1.6 (59) | 0.81 (93) | 4.7 (10) | 34.5 (62) | 8.9 (87) | 3.4 (183) | 2.6 (181) |
| *AA* | 2.9 (60) | 7.1 (125) | 4.4 (199) | 1.4 (80) | 9.3 (53) | 2.7 (158) | 0.45 (28) | 2.0 (58) | 0.92(100) | 6.3 (13) | 23.9 (58) | 5.5 (94) | 3.6 (359) | 2.2 (198) |
| ***OAT1* Rs4149170** | | | | | | | | | | | | | | |
| *AA* | 1.3 (7) | 6.5 (9) | 2.6 (18) | [c] | [c] | 2.6 (4) | 0.3 (16) | 1.2 (25) | 0.59 (47) | 1.8 (4) | 26.5 (37) | 6.7 (51) | 2.6 (22) | 2.1 (98) |
| *GA* | 2.4 (35) | 6.1 (57) | 4.1 (93) | 1.1 (18) | 9.0 (25) | 3.5 (49) | 0.3 (24) | 2.6 (64) | 1.1 (102) | 7.4 (14) | 33.0 (65) | 8.4 (100) | 3.9 (142) | 3.0 (207) |
| *GG* | 3.3 (64) | 6.7 (135) | 4.3 (215) | 1.4 (92) | 9.7 (65) | 2.9 (186) | 0.7 (12) | 1.8 (48) | 1.1 (72) | 5.1 (6) | 28.0 (44) | 7.5 (64) | 3.6 (203) | 2.7 (136) |
| ***OAT3* Rs4149182** | | | | | | | | | | | | | | |
| *CC* | 2.4 (8) | 12.8 (10) | 5.0 (19) | 1.7 (6) | 9.5 (4) | 3.0 (11) | 0.33 (17) | 1.2 (25) | 0.58 (48) | 1.8 (4) | 25.9 (39) | 7.0 (53) | 4.2 (30) | 2.1 (101) |
| *CG* | 3.5 (42) | 5.6 (64) | 3.7 (116) | 1.0 (30) | 6.8 (35) | 2.7 (81) | 0.34 (23) | 2.5 (66) | 1.1 (105) | 6.9 (14) | 32.0 (64) | 8.1 (99) | 3.2 (198) | 2.9 (209) |
| *GG* | 2.4 (56) | 6.9 (125) | 4.4 (190) | 1.4 (76) | 11.4 (52) | 3.1 (150) | 0.91 (11) | 1.9 (46) | 1.2 (67) | 6.2 (6) | 28.4 (43) | 7.5 (63) | 3.7 (341) | 2.9 (230) |

[a]Numbers of individuals is within brackets.

[b]Control subgroups are not shown separately, but are included in the "All" group.

[c]Denotes that the genotype is pooled with the heterozygotes due to a low number of individuals (3 or less).
